# An Unusual Opportunity

**Published:** 1887-11

**Authors:** 


### AN UNUSUAL OPPORTUNITY.

We desire to call attention to the publisher's announcement, which will be found opposite the first page of this number. We believe that this opportunity to obtain a work that involved great labor and expense in its preparation is a rare one. "The Microscopic Structure of a Human Tooth," which is offered as a premium for subscriptions to this Journal, is one of the most beautiful works of the kind that has been issued from any press, in any country. It is in the form of a portfolio, sixteen and one-half by twelve inches, the cover being imitation of alligator's skin. There are twelve plates, drawn in India ink and exquisitely engraved, each covering a full page of the same size as the cover. They represent the different tisues of the tooth in such a manner that the merest tyro cannot fail to comprehend the intimate anatomy of that organ, and each plate has an explanatory text giving a full description.

Plate I shows a molar tooth in perfect contour, exhibiting the morphological character of the tooth.

Plate II is a working diagram of a tooth, locating the various tissues, with reference figures to the explanatory text.

Plate III is a longitudinal section of an incisor tooth, showing the structure of all the tissues.

Plate IV is the same section of an inferior molar tooth.

Plate V is a transverse section of the root of a bicuspid.

Plate VI is the blood-vessels of an injected pulp. It is impossible adequately to describe the beautiful system of arteries, veins and capillaries, so truthfully illustrated in this plate.

Plate VII is a section of a root parallel to the dentinal canaliculi, and showing the lacunæ and the lamellæ of cementum.

Plate VIII shows the living parts of a tooth, commencing with the odontoblasts.

Plate IX represents the odontoblasts, enamel rods, interglobular spaces and lamellated dentine.

Plates X, XI and XII are representations of peculiar forms and conditions of teeth.

C. H. Stowell, M. D., F. R. M. S., professor of Histology and Microscopy in the University of Michigan, formerly editor of *The Microscope,* and the author of a number of works on microscopy and histology, spent a great deal of time in perfecting the drawings for this work, and in preparing the text. It is published at the price of Six Dollars, and it will not be sold singly for less. By securing a large number of copies, the publishers of this Journal are enabled to offer it, in connection with a subscription, for a mere nominal sum. This is done for the purpose of placing the work in the hands of every dentist who desires it, and incidentally to help the subscription list of the Independent Practitioner. Of course every one will be aware of the fact that not only is there no money made upon it, but that some one sustains a pecuniary loss by the liberality of the offer.

If there be a dentist who is not entirely familiar with the various parts of a tooth, who, when he attends dental meetings or reads dental journals and hears or sees referred to the minute histological structure of the teeth has not a clear idea of what is referred to, he should obtain this portfolio atlas, where he will find the whole delineated. If a dentist desires to refer to these tissues, and wishes to make the whole matter plain to his hearers, he needs these large and beautiful illustrations. We have used it in college lectures, and have found nothing which so graphically illustrated that which we wished to make clear to students.

We have described the work at length, because we wish all to have a clear apprehension of what is offered them. The terms upon which it may be procured are announced in the prospectus of Vol. IX of this Journal.

